# 

**Published:** 1895-01

**Authors:** 


### CONTENTS FOR JANUARY, 1895.

ORIGINAL COMMUNICATIONS. PAGE.

Surgery of the Accessory Sinuses. By Geo. F. Cott, M. D. .... 321
The Home Treatment of Hay Fever. By A. L. Hall, M. D. .... 328
The Pathology of Trichinosis. By Frank J. Thornbury, M. D. .... 332
Atonic Dyspepsia. By A. L. Benedict, A. M., M. D. .... 334
Constitutional Treatment of Diseases of the Female Sexual Organs. By William Redin Kirk, M. D. .... 341

CLINICAL LECTURE.

Idiocy. By J. W. Putnam, M. D. .... 344

REPORTS OF SOCIETIES.

The Eighth Periodical International Ophthalmological Congress .... 346
Buffalo Academy of Medicine .... 356

SELECTION.

Kidney of Pregnancy .... 358

EDITORIAL.

A National Public Health Establishment .... 359
The Care of the Poor .... 360
Echoes of the Hot Springs Meeting .... 361
Topics of the Month .... 363

Personal .... 368

Obituary .... 369

Society Meetings .... 370

Book Reviews .... 372

Literary Notes .... 382

